# P300 Detection Based on EEG Shape Features

**DOI:** 10.1155/2016/2029791

**Published:** 2016-01-10

**Authors:** Montserrat Alvarado-González, Edgar Garduño, Ernesto Bribiesca, Oscar Yáñez-Suárez, Verónica Medina-Bañuelos

**Affiliations:** ^1^Graduate Program in Computer Science and Engineering, Universidad Nacional Autónoma de México, 04510 Mexico City, Mexico; ^2^Department of Computer Science, Instituto de Investigaciones en Matemáticas Aplicadas y en Sistemas, Universidad Nacional Autónoma de México, 04510 Mexico City, Mexico; ^3^Neuroimaging Laboratory, Department of Electrical Engineering, Universidad Autónoma Metropolitana, 09340 Mexico City, Mexico

## Abstract

We present a novel approach to describe a P300 by a shape-feature vector, which offers several advantages over the feature vector used by the BCI2000 system. Additionally, we present a calibration algorithm that reduces the dimensionality of the shape-feature vector, the number of trials, and the electrodes needed by a Brain Computer Interface to accurately detect P300s; we also define a method to find a template that best represents, for a given electrode, the subject's P300 based on his/her own acquired signals. Our experiments with 21 subjects showed that the SWLDA's performance using our shape-feature vector was 93%, that is, 10% higher than the one obtained with BCI2000-feature's vector. The shape-feature vector is 34-dimensional for every electrode; however, it is possible to significantly reduce its dimensionality while keeping a high sensitivity. The validation of the calibration algorithm showed an averaged area under the ROC (AUROC) curve of 0.88. Also, most of the subjects needed less than 15 trials to have an AUROC superior to 0.8. Finally, we found that the electrode C4 also leads to better classification.

## 1. Introduction

The P300 is an event-related potential (ERP) endogenous component that has a positive deflection that occurs in the scalp-recorded electroencephalogram (EEG) and typically elicited approximately 300 ms after the presentation of an infrequent stimulus (such as visual, auditory, or somatosensory) [1]. The specific set of circumstances for eliciting a P300 is known as the Oddball Paradigm which consists of presenting a target stimulus amid more frequent standard background stimuli. Under this paradigm, a P300, among other ERPs, is unconsciously elicited every time a subject's brain detects the target stimulus (the rare event). In fact, the P300 is a reasonable input signal, with desirable properties and stability to control Brain Computer Interfaces (BCI) [2], applications requiring precise real-time detection as well as memory and computation optimization [3, 4]. The feature vector dimensionality reduction has been a popular choice to achieve these goals within the BCI community because it decreases the complexity of classifiers [5].

The features of a P300 have been represented in time, frequency, time-frequency, and shape domains by using, among others, Wavelet Transform [6], Genetic Algorithms [7], and Common Spatial Patterns [8]. Additionally, the approaches more commonly used for P300 classification are Linear Discriminant Analysis, Stepwise Linear Discriminant Analysis [9], and Support Vector Machines [10].

In this work, we are interested in the shape domain because we assume that (i) every subject produces P300 signals whose waveform can be consistently represented by template curves and (ii) such template curves from a subject are more similar to curves with a P300 than to curves produced by EEG background activity [11]. Most techniques based on these ideas are classified into Cross Correlation Alignment (e.g., Woody's [12] and Maximum Likelihood (ML) [13] methods), Dynamic Time Warping (DTW) alignment [14, 15], and linear methods such as coherent averaging [11]. Although the latter is the most controversial of all, it is the fastest and the most commonly used averaging method because of the following argument: a P300 can be considered as a well-defined component since the alignment of its peaks “is most likely linear even though the distortion is nonlinear” [16]. For this reason, it is common practice to repeat the stimulation procedure to improve its signal-to-noise ratio (SNR) by coherently averaging several segments of filtered EEG signals generated after the stimulation (i.e., trials); the number of stimulations may vary from subject to subject for reasons explained in [17]. Coherent averaging implies that ERP components are unaffected by the averaging procedure and that any variability is due to noise [18]. However, P300's amplitude, latency, and waveshape vary not only between electrodes but also in time. The first variation is due to its position; that is, the farther the electrode is from the cortical area, the lower the amplitude is. Thus, if we average all the electrode signals without taking into account the latter consideration, we will damage the P300's properties; for this reason, usually, the electrode signals are processed individually. The variation in time is due to either biological determinants (e.g., increasing difficulty in perception and cognition of a task), subject's attention level, or experimenter-dependent variables [17]. Thus, the coherent average does distort most ERP's components [15, 19]; however, for a given subject, the averaged P300 remains consistent [20]. The previous considerations can be summarized in the following statement by Knuth et al. [18]: “Of course, waveshape variability also exists, but robust single-trial amplitude and latency estimates are nonetheless obtainable with the assumption of fixed component waveshapes.”

The novelty of this paper consists in the detection of P300 trials based on using pattern recognition techniques on its shape, represented by a feature vector. Specifically, we use a contour representation based on an adapted version of the Slope Chain Code (SCC) and some of its properties (e.g., the tortuosity measure) [21], as well as some general descriptors, such as the differences of areas, to describe the differences between curves. Importantly, chain codes have been successfully used to describe and classify other biosignals such as electrocardiograms [22]. The advantages of using the SCC are as follows: (i) it is self-contained, which implies that a chain does not need decoding, and (ii) it is finite, which means that the resulting chains can be classified using either grammatical techniques, syntactic analysis [23], or algebraic operations. Because the SCC is very expensive, we adapted it to make it computationally less demanding. In addition to the adapted SCC, we also present an offline calibration algorithm that reduces the dimensionality of the shape-feature vector, the number of subject's stimulations, and the number of electrodes needed by a BCI to accurately detect a subject's P300.

We organized the paper as follows. In Section 2, we define the shape-feature vector and explain the details of the proposed algorithm. Then, in Section 2.3, we present our methodology to set the Oddball Paradigm and the experiments to define the parameters needed for the proposed algorithm. In Section 3, we present key results and a discussion of the experiments designed to evaluate the classification performance. Finally, in Section 4, we provide some conclusions.

## 2. Materials and Methods

In this section, we describe the features of the ERP's waveform that we use as the vector of characteristics. Additionally, we present an offline calibration algorithm that reduces the dimensionality of the shape-feature vector, the number of trials for a subject, and the number of electrodes needed by a BCI to detect a subject's P300.

### 2.1. Feature Vector Based on ERP's Waveform

As we mentioned before, the vector of characteristics obtained from the waveform of a P300 is central to our work. A first step towards producing such a vector is the coherent averaging of a set of trials.

#### 2.1.1. Coherent Averaging

It is a well-known fact that coherent averaging increases the SNR in signals and we take advantage of this fact to enhance the small amplitude signals immersed in an EEG. We and other groups [25] assume that the coherent averaging is feasible because (i) there is no correlation between the ERP signal and the rest of the EEG, (ii) the stimulation time and the response reflected in the EEG signal are known, (iii) there exists a consistently detectable component (e.g., a P300), and (iv) the EEG is a random signal with zero mean.

In a common BCI experiment, a number of electrodes are used to acquire EEG signals. We refer to this number as *C*. The signal from an electrode is acquired *K* times (i.e., *K* trials). We will refer to the resulting set of all acquired signals (i.e., *K* signals for *C* electrodes) as *𝔼* and we divide it into two nonoverlapping subsets *𝕋* and *𝕍*. We use the set *𝕋* to train the calibration algorithm (which is discussed in Section 2.2) and the set *𝕍* to validate its performance (see Section 3.2). Furthermore, every EEG signal recorded by an electrode is discretized by *T* number of samples. Consequently, the *T*-dimensional vector representing an EEG signal can be represented as follows:

(1) $$\begin{array}{c} e=g+n, \end{array}$$

where **g** and **n** are also vectors representing the ERP signal and the EEG background (associated with the rest of the brain's activity), respectively. By coherently averaging the *K* signals of a single electrode, we have

(2) $$\begin{array}{c} \mu \left(\;E,K\;\right)=\frac{1}{K}\overset{K}{\underset{k=1}{\sum }}e_{k}=\bar{g}+\bar{n}=\frac{1}{K}\overset{K}{\underset{k=1}{\sum }}\left(\;g_{k}+n_{k}\;\right). \end{array}$$

In practice, the averaged vector $\bar{n}$ is considered to be the zero vector (that vector whose element values are all equal to zero) because the EEG is a random signal with zero mean with little autocorrelation.

Because we intend to use the waveform of the recorded ERP signals to generate the vector of features, we represent a recorded signal **e** as the following sequence of ordered pairs [(*x*′, *y*′)_1_, (*x*′, *y*′)_2_,…, (*x*′, *y*′)_*T*_], where *x*′ is a nonnegative integer corresponding to the sample number and *y*′ is a real number representing the measured amplitude of the ERP at the position *x*′. As a result, the coherent average of (2) produces the vector ${\bar{e}}^{\prime }=\left[{\left(x^{\prime },\bar{y}\right)}_{1},{\left(x^{\prime },\bar{y}\right)}_{2},\ldots ,{\left(x^{\prime },\bar{y}\right)}_{T}\right]$.

#### 2.1.2. Slope Horizontal Chain Code

Chain codes are alphanumeric sequences with integer alphabets being the most common choices because the easiness and velocity to process the resulting chains in comparison to those based on alphanumeric alphabets. Several integer-alphabet chain codes have been proposed [21, 26–32] as well as methods to represent analog signals with sequences of bits (e.g., pulse code modulation [33]); however, the SCC is the most useful for the purposes of this paper because it divides the curve into straight-line segments placed onto the curve and preserves with higher resolution the contour shape. By using the ordered-pair representation for ERP signals, we can obtain a chain code representing the contour of the curve described by its sequence of ordered pairs [34].

In this work, we adapted the SCC to represent ERP signals and called it Slope Horizontal Chain Code (SHCC). The main differences between the SCC and our code are the following. The SHCC adjusts a segment's length to avoid interpolation; this adjustment takes advantage of the sampling uniformity during the biosignal acquisition to keep the sampling points as the endpoints of segments. Contrary to the SCC, the SHCC does not compute the angle between two adjacent segments; in contrast, it computes the slope between a segment and the horizontal in the continuous range equivalent to (−90°, 90°). Consequently, the segments are independent, which means that if the signal from one electrode is disturbed (e.g., due to noise or loss of information), this will not affect more than one chain element. Furthermore, the SHCC does not require either rotation invariance, since it is not designed for closed curves, or scale invariance. Consequently, the previous differences make the SHCC algorithm computationally less expensive and very useful for real-time applications. Moreover, the SHCC can be easily implemented in hardware; thus, allowing the classifier integration to signal acquisition devices.

On the other hand, the SHCC and the SCC share the following very useful properties for our application: both place line segments onto the curve to preserve with high resolution the contour shape, both are translation-invariant, which is relevant since the SHCC can adequately represent P300's variability, and both allow feature dimensionality and data reduction. The two are very desirable properties in BCI applications [35].

A first step to transform the curve into a chain by the SHCC is to resample the vector ${\bar{e}}^{\prime }$ with a new sampling distance given by

(3) $$\begin{array}{c} \Delta =\left\lceil \;\frac{T}{S+1}\;\right\rceil , \end{array}$$

where *S* < *T* is a nonnegative integer representing the desired number of line segments to represent the curve (in Section 2.3.4, we will explain the procedure to select the *S* value). The new rediscretized vector is a sequence of ordered pairs $\left[{\left(x^{\prime },\bar{y}\right)}_{1},\ldots ,{\left(x^{\prime },\bar{y}\right)}_{s},\ldots ,{\left(x^{\prime },\bar{y}\right)}_{S}\right]$, where *s* = ⌊*t* + (*m* × Δ) + 0.5⌋, for 1 ≤ *t* ≤ *T*, and *m* = sgn⁡((*T* − 1)/Δ)⌊|(*T* − 1)/Δ|⌋. An alternative to this rediscretization process would be to change the sampling rate (i.e., subsampling) during the acquisition process but this can potentially distort the ERP signal, due to aliasing, and produce regions of the signal similar to a P300, which in turn could produce false positives in the classification stage.

Before obtaining the alphabet symbols, the SHCC normalizes every element $\left(x^{\prime },\bar{y}\right)$ of ${\bar{e}}^{\prime }$ as follows:

(4) x=1maxxix′−minxix′x′−minxi⁡x′1,y=1maxyiy¯−minyiy¯y¯−minyi⁡y¯1,

where** 1** is a vector whose element values are all equal to one.

**Figure 1:**
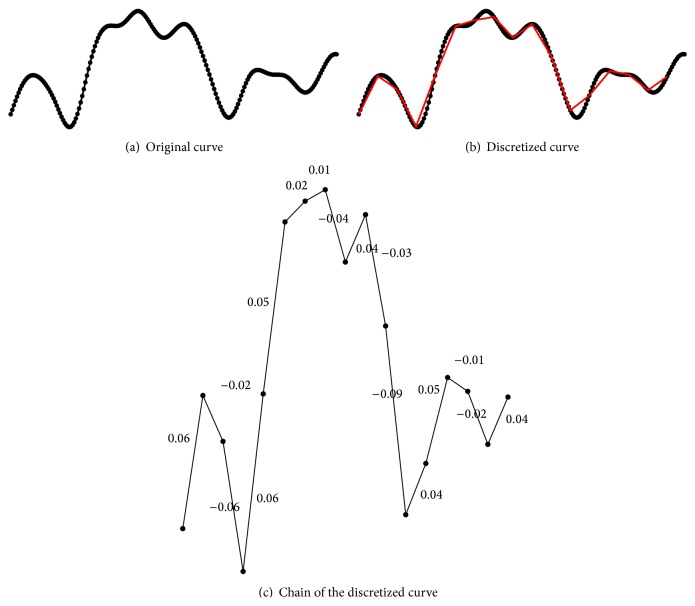
Example of a P300 discretized curve and its resulting chain code by using the SHCC method.

These operations produce new coordinate vectors **x** = [*x*_1_,…, *x*_*S*_] and **y** = [*y*_1_,…, *y*_*S*_], where *x*_*i*_, *y*_*i*_ ∈ [0,1]. With these coordinates, the SHCC produces a chain *ℬ* = (*b*_1_ … *b*_*S*_) whose *s*th element represents the code associated with the slope between the horizontal axis and the *s*th ordered pair (*x*, *y*)_*s*_, for 1 ≤ *s* ≤ *S*. To compute the members of the alphabet, we use a precision of two decimals when computing the individual slopes, resulting in an alphabet of 200 elements. To exemplify this process, we show in Figure 1 a discretized ERP whose chain is *ℬ* = (0.06 −0.02 −0.06 0.06 0.05 0.02 0.01 −0.04 0.04 −0.03 −0.09 0.04 0.05 −0.01 −0.02 0.04).

Finally, to form a vector of characteristics, we consider the elements of a chain obtained with the aforementioned SHCC method as part of the features of the vector together with other characteristics as we will show below.

#### 2.1.3. Distance between Chains

The possibility of computing the distance between two curves is an important characteristic that we take advantage of for our proposed method. Since we use the SHCC to represent 2D curves, we obtain a unique curve descriptor represented by a chain. The hypothesis is that the chain representing a P300 template is more similar to the chain of a P300 than to the chain from a non-P300.

There are several distances to measure shape dissimilarity for 2D curves such as the Manhattan (i.e., the *ℓ*_1_-norm), the Euclidean (i.e., the *ℓ*_2_-norm), the Hausdorff, or the Frèchet distances [36]. To decide between them, we ran experiments with preliminary parameters to compare our algorithm's performance (we explain our algorithm below) and the results were not significantly different; thus, we decided to compute the distance with the *ℓ*_1_ norm because of its lower computational cost. Consequently, for two chains *ℬ*_*i*_ and *ℬ*_*j*_ of length *S*, we define their distance *d* as

(5) $$\begin{array}{c} d=l_{1}\left(\;B_{i},B_{j}\;\right)=\overset{S}{\underset{s=1}{\sum }}\left|\;b_{i,s}-b_{j,s}\;\right|. \end{array}$$

**Figure 2:**
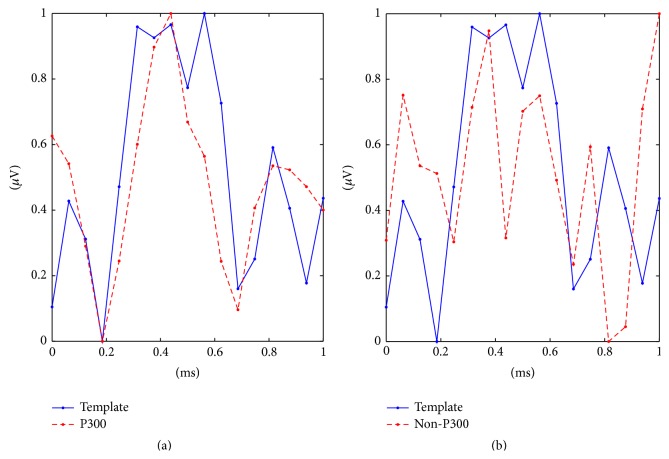
Illustration of the difference between a subject's template curve and (a) a P300 curve and (b) a non-P300 curve.

To exemplify our hypothesis, we show different subsampled curves in Figure 2. In Figure 2(a), we present in blue the template curve of a subject, whose chain is (0.05 −0.02 −0.05 0.08 0.07 −0.01 0.01 −0.03 0.04 −0.04 −0.09 0.01 0.05 −0.03 −0.04 0.04), and in red a P300 curve of the same subject, whose chain is (−0.01 −0.04 −0.05 0.04 0.05 0.05 0.02 −0.05 −0.02 −0.05 −0.02 0.05 0.02 0 −0.01 −0.01). The Manhattan distance between these two chains is 0.55. In contrast, in Figure 2(b), we present the same template curve together with a non-P300 curve, whose chain is (0.07 −0.03 0 −0.03 0.06 0.04 −0.10 0.06 0.01 −0.04 −0.04 0.06 −0.09 0.01 0.11 0.05); in this case, the distance between chains is 0.92. For this example, one can see that the subject's template curve is more similar to the P300 curve than to the non-P300 curve. This is just an example and within the calibration process there is some statistical test that makes sure that this hypothesis is met.

#### 2.1.4. Tortuosity

Another feature that we would like to capture is how straight or twisted a curve is; one way to measure such a characteristic is by

(6) $$\begin{array}{c} \Upsilon \left(\;B\;\right)=\overset{\left(S-1\right)}{\underset{i=1}{\sum }}\left|\;\frac{\left\lfloor b_{i}\right\rfloor }{100}\;\right|, \end{array}$$

where *b*_*i*_ is the *i*th element of the chain *ℬ*. The minimum value of this measure is zero, corresponding to a curve consisting of purely horizontal segments (i.e., all the components have slopes equal to zero). On the other hand, as the curvature increases, the value of *Υ* will also increase [21]. The measure *Υ* above is commonly known as* tortuosity*, and, for example, the tortuosity value of the curve shown in Figure 1 is 0.64.

#### 2.1.5. Differences between the Areas of Two Curves

Both the SCC and the SHCC describe the signals waveform at the expense of losing voltage information; the latter is useful for discrimination between conditions. Moreover, two curves having different shape could have the same tortuosity. For these reasons, we introduce an additional way to compare two curves by computing the difference between their areas. To this end, we apply the trapezoidal rule [37], because it integrates a curve over an interval by breaking the area under the curve into small trapezoids whose areas are easier to compute. In what follows, for a subject, we will compute the difference between the areas of a template curve (*ℛ* ^*T*^) and the area of either a P300 curve (*ℛ* ^*P*^) or a non-P300 curve (*ℛ* ^*N*^). We refer to the segment-wise differences as [*a*_1_, *a*_2_,…, *a*_*S*−1_]. For any two *S*-dimensional vectors **p** and **q**, we define the sum of their segment-wise differences as

(7) $$\begin{array}{c} \overset{˘}{T}\left(\;p,q\;\right)=\overset{\left(S-1\right)}{\underset{s=1}{\sum }}p_{s}-q_{s}. \end{array}$$

Finally, for every electrode, we assemble a (2*S* + 2)-dimensional vector of shape features by combining all the described parameters above in the following way: the first (*S* − 1) elements of the vector correspond to the differences between the area of two curves [*a*_1_, *a*_2_,…, *a*_*S*−1_], the following element is the sum of them $\overset{˘}{T}$ by (7). The next element is the distance between chains *d* by (5), followed by the tortuosity measure *Υ* by (6), and the last *S* elements of the vector represent the ERP under analysis (*b*_1_,…, *b*_*S*_), resulting in the vector $v=\left[a_{1},a_{2},\ldots ,a_{S-1},\overset{˘}{T},d,\Upsilon ,b_{1},b_{2},\ldots ,b_{S}\right]$ of size *V*.

### 2.2. Calibration Algorithm

Like any other BCI system that uses a feature vector, we need to calibrate ours for every subject. For our project, the goals of the calibration are (i) to obtain a template for every electrode that best represents a subject's P300 in that electrode, (ii) to obtain the optimum number of stimulations, (iii) to select the subset of electrodes that provides the best P300 signal, and (iv) to select the shape features that maximize the* area under the receiver operating characteristic* (AUROC)* curve.*

In what follows, we define some sets and variables necessary for our calibration algorithm. For the calibration process, we select a certain number of P300-labeled trials and a certain number of non-P300-labeled trials for a given electrode *c*. We refer to the set of non-P300-labeled trials, with cardinality equal to *N*, as *ℰ*_*c*_^*N*^ and as *ℰ*_*c*_^*P*^ to the set of P300-labeled trials, with cardinality equal to *P*. Clearly, the total number of trials, for a given electrode *c*, produces a set; we refer to it as *ℰ*_*c*_ (i.e., *ℰ*_*c*_ = *ℰ*_*c*_^*P*^ ∪ *ℰ*_*c*_^*N*^). When all the electrodes that we use are taken into account, then the set of all the trials results in the set *𝕋* which can then be expressed as {*ℰ*_*c*_∣1 ≤ *c* ≤ *C*}.

Algorithm 1 presents the pseudocode for our calibration method, which is an iterative algorithm based on* wrapper* methods [38].

A general view of the calibration algorithm is as follows. The iterative algorithm is made of several sections that carry a specific task and are called* wrappers*. The principal* wrapper* is an iterative procedure; see lines 4–24. To control the iterations, we make use of the boolean variable* loop* initialized in line 1. The algorithm iterates while the value of* loop* is true (see line 4). The goals of this* wrapper* are to select (i) the electrodes and the shape features that provide the best P300 signal, (ii) the best templates for each electrode, and (iii) the optimum number of stimulations. We select the subset of electrodes and the optimum number of stimulations by finding the templates that satisfy certain criteria. The number of trials (i.e., *K*) is one of the parameters defined in the experimental design (see Section 2.3.3). We define the variable *k* as the maximum number of stimulations *K* (see line 2), which will gradually decrease to diminish subject's fatigue, to find the optimum number of stimulations. Additionally, we find the best templates and the best shape features by means of an inner* wrapper* method that iterates *O* number of times, where *O* is defined in line 5. In each iteration, the inner* wrapper* randomly selects a set of P300-labeled trials to find the best template and the best features for each electrode by analyzing subsets of trials. For this analysis to be statistically significant, we apply a cross-validation *U* times, where *U* is defined in line 3. Since *U* depends on *K*, it could be computed after *K* is set. Then, the inner* wrapper* evaluates the detection of a P300 through lines 6–19. On the other hand, in order to reduce the shape-feature vector dimensionality, we use the* stepwise regression method* (SRM) described in [5, 39]. This method performs feature space reduction by selecting the elements of the shape-feature vector that satisfy certain entry and removal criteria.

Now, we describe the inner* wrapper* in detail. As we explained before, for an electrode *c*, we need to search for the subject's best template that consistently represents a P300 waveform (see lines 7–18). Each template chain is generated by randomly selecting a subset *ℛ* ^*T*^ = {**e**_*i*_∣**e**_*i*_ ∈ *ℰ*_*c*_^*P*^  and  1 ≤ *i* ≤ *A*}, where *A* is the number of trials necessary to generate the subject's P300 template (we will detail the method to define both *A* and *S* in Section 2.3.4). We perform this task by the* RandomSelector* operator (see line 8). Then, we compute its coherent average *μ*(*ℛ* ^*T*^, *A*) by (2) and we transform the resulting vector into a chain *ℬ*_*o*,*c*_^*T*^ using the SHCC (see Section 2.1.2); this process is carried out in line 9 by the operator* ChainCodeSHCC*. At this point, the algorithm creates a set of several template chains {*ℬ*_1,*c*_^*T*^,…, *ℬ*_*o*,*c*_^*T*^,…, *ℬ*_*O*,*c*_^*T*^} for a given electrode *c*.

Every template chain *ℬ*_*o*,*c*_^*T*^ is compared with the chains *ℬ*_*c*_^*P*^ and *ℬ*_*c*_^*N*^, where *ℬ*_*c*_^*P*^ is the chain of the average subset *ℛ* ^*P*^, whose *k* elements (i.e., the number of stimulations) are randomly selected from the set {*ℰ*_*c*_^*P*^ − *ℛ* ^*T*^} (see lines 11 and 12); and *ℬ*_*c*_^*N*^ is the chain corresponding to the averaged subset *ℛ* ^*N*^, composed of *k* elements randomly selected from *ℰ*_*c*_^*N*^ (see lines 15 and 16). These comparisons are carried out (see lines 8–17) *O* times (see line 6) per electrode.

After these comparisons, we obtain two shape-feature vectors **v** ^*P*^ and **v** ^*N*^ as explained in Section 2.1. The vector **v** ^*P*^ represents the features extracted from *ℛ* ^*T*^, *ℛ* ^*P*^, *ℬ*_*o*,*c*_^*T*^, and *ℬ*_*c*_^*P*^ (see line 13); the vector **v** ^*N*^ represents the features extracted from *ℛ* ^*T*^, *ℛ* ^*N*^, *ℬ*_*o*,*c*_^*T*^, and *ℬ*_*c*_^*N*^ (see line 17); this process is performed by the operator* FeatureExtractor*. For this analysis to be statistically significant, we apply a cross-validation test. Hence, we compute vectors **v**_*u*,*c*_^*P*^ and **v**_*u*,*c*_^*N*^ *U* times (see lines 10 and 14, resp.) for each electrode. These vectors allow the creation of the 2*U* × *VC* matrix defined as

(8) $$\begin{array}{c} V_{o}=\left[\begin{array}{ccc} v_{1,1,1}^{P} & \cdots & v_{1,V,1}^{P}\\ v_{2,1,1}^{P} & \cdots & v_{2,V,1}^{P}\\ \vdots & & \vdots \\ v_{U+1,1,1}^{P} & \cdots & v_{U+1,V,1}^{P}\\ v_{1,1,1}^{N} & \cdots & v_{1,V,1}^{N}\\ v_{2,1,1}^{N} & \cdots & v_{2,V,1}^{N}\\ \vdots & & \vdots \\ v_{2U,1,1}^{N} & \cdots & v_{2U,V,1}^{N} \end{array}\begin{array}{ccc} v_{1,1,2}^{P} & \cdots & v_{1,V,\ldots }^{P}\\ v_{2,1,2}^{P} & \cdots & v_{2,V,\ldots }^{P}\\ \vdots & & \vdots \\ v_{U+1,1,2}^{P} & \cdots & v_{U+1,V,\ldots }^{P}\\ v_{1,1,2}^{N} & \cdots & v_{1,V,\ldots }^{N}\\ v_{2,1,2}^{N} & \cdots & v_{2,V,\ldots }^{N}\\ \vdots & & \vdots \\ v_{2U,1,2}^{N} & \cdots & v_{2U,V,\ldots }^{N} \end{array}\begin{array}{ccc} v_{1,1,\ldots }^{P} & \cdots & v_{1,V,C}^{P}\\ v_{2,1,\ldots }^{P} & \cdots & v_{2,V,C}^{P}\\ \vdots & & \vdots \\ v_{U+1,1,\ldots }^{P} & \cdots & v_{U+1,V,C}^{P}\\ v_{1,1,\ldots }^{N} & \cdots & v_{1,V,C}^{N}\\ v_{2,1,\ldots }^{N} & \cdots & v_{2,V,C}^{N}\\ \vdots & & \vdots \\ v_{2U,1,\ldots }^{N} & \cdots & v_{2U,V,C}^{N} \end{array}\right]. \end{array}$$

To evaluate the performance of the calibration process by using one template at a time, we decided to use the computed distances between chains as accuracy measures (based on preliminary experiments); this process is carried out in line 18 by the operator* AUROCEstimator*. For every *i*th column of matrix **V**_*o*_, we take the *S* + 1 element of the first *U* vectors to form a vector **d**_*c*_^*P*^, whose elements are the distance element of such vectors; in the same way, we take the *S* + 1 element of vectors *U* + 1,…, 2*U* to form a vector **d**_*c*_^*N*^. Thus, **d**_*c*_^*P*^ and **d**_*c*_^*N*^ are *U*-tuples (*d*_1,*c*_^*P*^, *d*_2,*c*_^*P*^,…, *d*_*U*,*c*_^*P*^) and (*d*_1,*c*_^*N*^, *d*_2,*c*_^*N*^,…, *d*_*U*,*c*_^*N*^). Then, we compute the AUROC to evaluate the comparisons between **d**_*c*_^*P*^ and **d**_*c*_^*N*^ and create a matrix entry *z*_*co*_. Each of these entries represents the discrimination capacity for the *o*th template of each electrode. These entries build a *O* × *C* matrix defined as

(9) $$\begin{array}{c} Z=\left[\begin{array}{cccc} z_{1,1} & z_{2,1} & \cdots & z_{O,C}\\ z_{1,2} & z_{2,2} & \cdots & z_{O,C}\\ \vdots & \ddots & & \\ z_{1,C} & z_{2,C} & \cdots & z_{O,C} \end{array}\right]. \end{array}$$

As mentioned earlier, one of the goals of the principal* wrapper* is to reduce the shape-feature vector dimensionality. To that end, we use the operator* Stepwise* (see line 19) that computes the SRM; its entry criteria is *P*-value < 0.1 and its removal criteria is *P*-value > 0.15. These *P*-values were defined based on those reported in [5]. The* Stepwise* renders the *I*-dimensional vector **f** of the *I* elements of **V**_*o*_ selected by the method SRM, the binary *I*-dimensional vector **i**, representing the indexes of the vector **f**, and the *I*-dimensional vector **w** of estimated coefficients for all the terms in **V**_*o*_. Every *j*th element of **w** whose corresponding *j*th element of **i** is different from zero will be an entry to the vector **w**_*o*_′. Likewise, every *j*th element of **i** whose value is different from zero will be an entry to the vector **i**_*o*_′. Finally, every element of **f** related to the *i*th element of **i**_*o*_′ will be an entry to the vector **f**_*o*_′. The latter procedure finishes the inner* wrapper*.

As we mentioned before, some goals of the principal* wrapper* are to select both the subset of electrodes that provides the best P300 signal and the optimum number of stimulations by finding the templates that satisfy certain criteria. To that end, the operator *averageAUROC* (line 20) computes the average AUROC to measure the performance of the templates of each electrode, for each stimulation *k* by

(10) $$\begin{array}{c} \phi_{k}=\frac{1}{O}\overset{O}{\underset{o=1}{\sum }}z_{o}. \end{array}$$

The operator *ElectrodeSelector* (see line 21) selects the subset of electrodes that provides the best P300 signal for each subject. To achieve this goal, we select the electrodes where *ϕ*_*k*,*c*_ ≥ 0.8. If there are no such electrodes, then we choose those where *ϕ*_*k*,*c*_ > 0.6 and set the variable* loop* to false. At the end, the algorithm will define as **c**′ the set of electrodes that meet these conditions; otherwise, it will be unsuitable to find the subject's P300. On the other hand, to select the subject' s optimum number of stimulations, *K* is gradually decreased until no more electrodes are found meeting the condition where *ϕ*_*k*,*c*_ ≥ 0.8. In such case, *K* − 1 will be the optimum number of stimulations *K*′, and the algorithm stops. This process is carried out by the operator *TrialSelector* (see line 22). The operator *TemplateSelector* (see line 23) selects the templates *ℬ*_*c*_′ = *ℬ*_argmax_1≤*o*≤*O*_(**Z**),*c*_^*T*^ that achieve the highest values of **Z** for each electrode of the set **c**′. Finally, the operator *FeatureSelector* (see line 24) selects the indexes **I**_*c*_′ = **i**_argmax_1≤*o*≤*O*_(**Z**),*c*_′ of the shape-feature vectors, the matrix of regression coefficients **W**_*c*_′ = **w**_argmax_1≤*o*≤*O*_(**Z**),*c*_′, and the features **F**_*c*_′ = **i**_argmax_1≤*o*≤*O*_(**Z**),*c*_′ associated with the previously selected templates. The algorithm returns the **c**′, *K*′, *ℬ*_*c*_′, **I**_*c*_′, **W**_*c*_′, and **F**_*c*_′ values that we store in a text file.

### 2.3. Experimental Design

#### 2.3.1. Participants

For our experiments, we used the EEG signal database as reported in [24], acquired by the Neuroimaging Laboratory (LINI) of the Universidad Autónoma Metropolitana (UAM), Iztapalapa. We used the EEG signals from 21 healthy students (8 females and 13 males) ranging in age from 21 to 25 years without known neurological conditions. They slept an average of 7.5 hours the night before the experiment. Four students smoked one cigarette 24 hours before the experiments and one of them smoked 5.

#### 2.3.2. Data Acquisition and Processing

**Figure 3:**
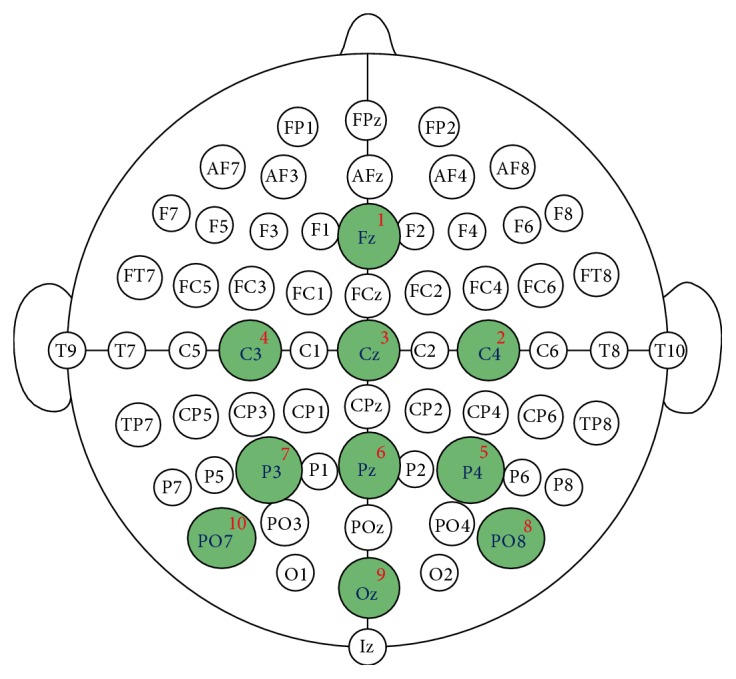
The electrode configuration used in the current study. The EEG was acquired by 10 electrodes located at the international 10-20 system. Image from [24] with author's permission.

The EEG-signal database was acquired using 10 electrodes denoted by Fz, C4, Cz, C3, P4, Pz, P3, PO8, Oz, and PO7 following the international 10-20 system (see the configuration in Figure 3), with the right earlobe and the right mastoid serving as reference and ground locations. For the acquisition, screwable passive Ag/AgCl EEG electrodes were used (g.EEGelectrode manufactured by GTec [40]). The impedances between the cap electrodes and the reference electrode never exceeded 5 kΩ. The EEG signals were registered and amplified using a 24-bit g.USBamp [41] amplifier. The signal was digitized at a rate of 256 Hz and processed online with a notch filter (Chebyshev of order 4), with cutoff frequencies between 58 and 62 Hz, and a bandpass filter (Chebyshev of order 8), with cutoff frequencies between 0.1 and 60 Hz, to reduce noise. All aspects of data collection and experimental design were controlled using the BCI2000 system [42].

#### 2.3.3. Task Description and ERP Signal Extraction

Despite the fact that our proposed method can be used in any BCI application, we decided to apply it to the P300 word speller, first described by [43], in order to make a comparison with a widely documented system. In the P300 word speller, a subject is presented with an alphanumeric matrix projected onto a computer screen to allow him or her to write a word. We did not include additional procedures that may bias the performance, for example, by inferring symbols.

The participants were asked to spell* a priori* known words from which we acquired 2,880 EEG signals to form the set *𝔼*. These signals were distributed into the training set (*𝕋*) and the test set (*𝕍*). The set *𝕋* consisted of 480 EEG signals that potentially contained P300s (i.e., *P* = 480) and 2,400 EEG signals without P300s (i.e., *N* = 2,400). The set *𝕍* contained 150 signals expected to have 150 P300s (i.e., *P*′ = 150) and 750 non-P300s (i.e., *N*′ = 750).

**Figure 4:**
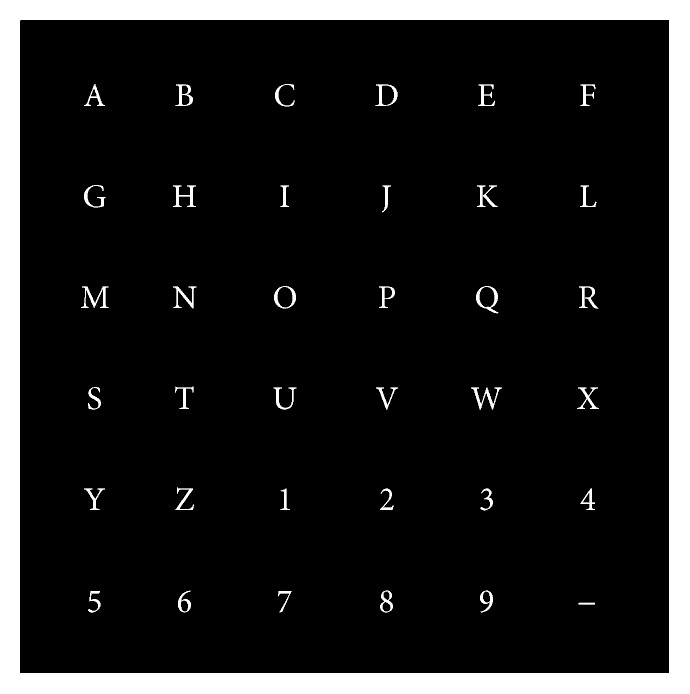
The 6 × 6 matrix speller used in the current study.

For the experiments, the participants sat in front of the computer screen, which is divided into two sections. At the top left corner of the screen, the word to be spelled was displayed while the character currently specified for selection was listed in parenthesis at the end of the word. The remaining of the screen displays a 6 × 6 matrix speller, as shown in Figure 4.

The matrix rows and columns were randomly intensified 15 times (i.e., trials) for every letter. The subjects were asked to silently count the number of times the target character was intensified while the matrix rows and columns flashed every 125 ms in random order (i.e., the interstimulus time); every flash lasted 62.5 ms. Because of the nature of the signal, we expected to have a P300 wave 300 ms after every stimulus. For this reason, we decided to extract the next 800 ms of EEG data after every stimulus per channel used in the analysis; thus, we collected around 2 P300 waves in every trial due to the interstimulus time. Each segment of 800 ms was filtered offline using a 4th-order Butterworth bandpass filter with bandwidth range from 0.1 to 12 Hz to extract the ERP signals embedded in the EEG as it is common in the field [44]. The DC component was removed by subtracting the mean of each electrode from the filtered signal. Finally, the linear trend was removed from each trial.

#### 2.3.4. Algorithm Parameters

Now, we explain the methodology used to select the parameters *S* and *A* required by the calibration algorithm. As we showed in (3), *S* represents the number of straight-line segments used to divide an ERP signal to obtain a minimal representation of its shape. Correspondingly, *A* is the number of a subject's P300 trials needed to accurately represent a template.

Our goal is to preserve the P300's envelope by the minimum representation possible while allowing its detection with the SHCC (see Section 2.1.2). To that end, we chose the value of *S* based on the fact that the ERP bandwidth can get up to 10 Hz [45] and that the sampling frequency must satisfy the Nyquist Theorem; thus, we decided on a resampling frequency of 20 Hz. Considering that we extracted trials of 800 ms, 16 segments were sufficient to preserve the waveshape (in other words, *S* = ⌊800 ms × 20 Hz/1000 ms⌋ = 16) and the maximum value of *S* (without signal interpolation) is equal to *T* − 1. With this value in consideration, the shape-feature vector **v** is 34-dimensional for every electrode.

**Figure 5:**
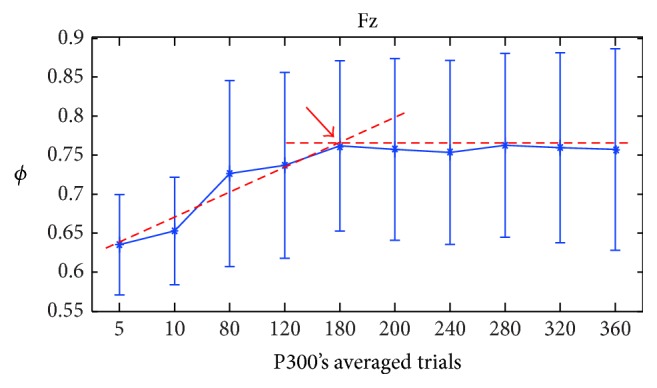
Behavior of the calibration AUROC while running several calibration experiments with a fixed *S* = 16 and varying the number of P300 averaged.

On the other hand, to determine the number of trials necessary for a template to accurately represent a subject's P300 for each electrode, we ran experiments with the calibration algorithm varying the values of *A* with the arbitrarily chosen values {5,10,80,120,180,200,320,360}. For these experiments, we fixed the value of *S* to 16, in view of the discussion in the previous paragraph, and the value of *K* to 15 trials. Then, we computed the mean and the standard deviation of *ϕ* for all subjects and for every electrode (see Figure 5). For a better interpretation, in this figure, the ordinate represents the amount of P300 necessary to average while the abscissa indicates the *ϕ* measure. From these results, we selected a value for *A* where an inflection point is reached in most of the electrodes, in this case around the value of 180 (i.e., *A* = 180).

## 3. Results and Discussion

In this section, we report and discuss the design and results of our experiments; they test the performance of our method in detecting P300 trials based on its shape-feature vector.

### 3.1. Calibration Algorithm

In order to test the performance of the calibration algorithm, we fixed the values for parameters *A* and *S* to 180 and 16, respectively, as we explained in Section 2.3.4; we also fixed the number of electrodes *C* to 10 and the number of stimulations *K* to 15, as defined in Section 2.3.2.

**Figure 6:**
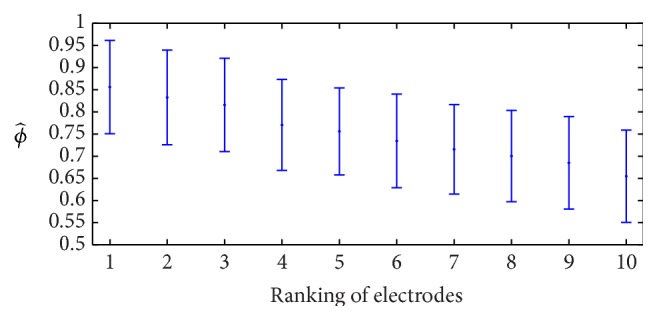
Ranking of electrodes according to mean *ϕ*, where electrode 1 corresponds to the best performing electrode for each subject (not necessarily the same), while electrode 10 is the one with the lowest *ϕ* per subject.

We performed the cross-validation of the Calibration Algorithm with the training dataset *𝕋*. We set the value *U* (see line 3) to *U* = ⌊(*P* − *A*)/*K*⌋ = ⌊(480 − 180)/15⌋ = 20. That is, we randomly selected 20 P300-labeled signals and 20 non-P300-labeled signals to balance the datasets. For each subject, we obtained a mean AUROC *ϕ* computed by (10). The average for the studied population $\left(\hat{\phi }\right)$ is presented in Figure 6, where electrode 1 corresponds to the best performing electrode for each subject (not necessarily the same), while electrode 10 is the one with the lowest *ϕ* per subject. In this figure, we can observe that the average value for electrode 1 for all subjects was 0.87 ± 0.10. Among all the subjects, two of them had one electrode with *ϕ* close to one (0.99 ± 0.002 and 0.99 ± 0.01); 12 of them had at least one electrode with *ϕ* ≥ 0.9; 16 subjects had at least one electrode with *ϕ* ≥ 0.8; and 20 subjects had at least one electrode with *ϕ* ≥ 0.7. The worst case was one subject whose best *ϕ* was equal to 0.64 ± 0.14, possibly because the subject was distracted for several reasons such as fatigue, lack of motivation, or hunger [17].

It is worth noting that the normalization process performed by the SHCC could be sensitive to outliers. However, the filters and the subsampling we applied to the signal reduce outliers. Moreover, the AUROCs reflect an adequate performance, even with a nonoptimal normalization.

On the other hand, it is common practice to stimulate a subject fifteen times for every letter [46]. However, our experiments suggest that P300s can be accurately detected with fewer than fifteen stimulations with our calibration algorithm (see Table 1); however, this observation is subject-dependent: fourteen of the twenty-one subjects required fewer than fifteen stimulations to have at least one electrode which had a mean AUROC greater than or equal to 0.8. Moreover, eight of them needed at most five stimulations, and in one case the subject only required two.

**Figure 7:**
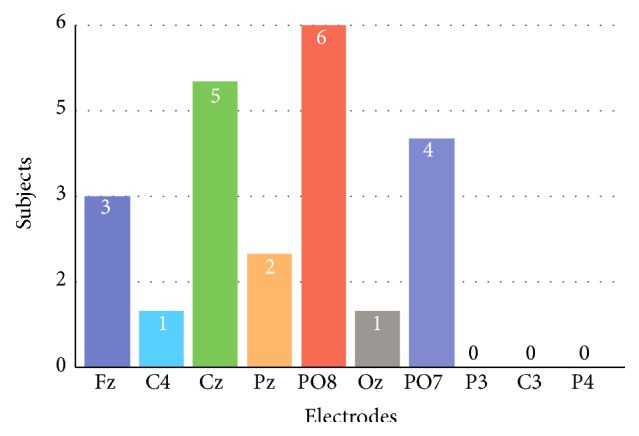
Incidence of subjects whose mean AUROC during the calibration process was greater than or equal to 0.8 when they are stimulated an optimum number of times (see Table 1).

Additionally, we analyzed the behavior of the sets of electrodes for all subjects based on calibrations *ϕ*'s. For this purpose, we carried out two experiments. In the first one, we set the number of stimulations *K* to 15. The incidence of subjects whose electrodes provided a *ϕ* ≥ 0.8 is as follows. The PO8 electrode met this criterion with 27% (corresponding to six subjects), followed by Fz with 18% (corresponding to four subjects), Cz and PO7 with 14% (corresponding to three subjects), and C4, Pz, and Oz with 9% (corresponding to two subjects). The electrodes that did not meet this criterion were P3, C3, and P4. In the second experiment, we expected the optimum number of stimulations shown in Table 1 to perform as the previous experiment.  Figure 7 illustrates the incidence of subjects whose electrodes provided a *ϕ* ≥ 0.8. The electrodes PO8 and Cz had the highest incidence of all, 27% (corresponding to six subjects) and 23% (corresponding to five subjects), respectively, followed by PO7 and Fz, with 18% (corresponding to four subjects) and 14% (corresponding to three subjects). Finally, the electrodes Pz, Oz, and C4 had the lowest incidence with Pz having only 9% (corresponding to two subjects) and both Oz and C4 having 5% (corresponding to one subject), the remaining two.

We have hypothesized that the discrepancy between the incidences shown above is the result of the criterion that we use for selecting the electrodes. Certainly, there can be other criteria for this selection like statistical tools such as a *t*-test to decide whether an electrode is really better than another.

After carrying out the selection of electrodes, we found out that the best information is provided by electrodes C4, Cz, Fz, Pz, PO7, PO8, and Oz, unlike the P3, C3, and P4 electrodes whose information can be discarded since they did not contribute to a desired performance. These results are consistent with the literature [5, 47] which claims that the P300 is generally detected in the central, frontal, parietal, and parieto-occipital areas of the scalp (i.e., in the electrodes Cz, Fz, Pz, PO7, PO8, and Oz), regions associated with attention, memory, and visual processes. Additionally, we observed that C4 also provides the best performance for several subjects. For this reason, we suggest using the aforementioned electrodes for speller P300 experiments as a common practice. Therefore, the calibration algorithm could be useful to discard those electrodes that do not provide a relevant information for our methods, either because they are improperly placed, which would generate an undesirable template, or because they will lead to a misclassification. Moreover, if signals are obtained by electrodes other than the ones we suggested, the calibration algorithm will be able to select the ones that lead to the best performance of the calibration process.

### 3.2. Validation

The aim of the validation process is to analyze the performance of the P300 detection when using our shape-feature vector **v**, the set of templates found for the selected electrodes, and the optimum number of stimulations obtained by the Calibration Algorithm. To that end, we generated the following two experiments. In the first experiment, we compared the performance of two classifiers commonly used by the BCI community [9]: the* Stepwise Linear Discriminant Analysis* (SWLDA) and* Support Vector Machine* (SVM) by using the vector **v**. In the second experiment, we compared the performance of SWLDA (considered as one of the best BCI classifiers by Krusienski et al. [9]) by applying both vectors **v** and the one used by the BCI2000 system (**v**′) as described in [5].

In the first experiment, we extracted a balanced subset *𝔹* from the training set *𝕋*. The set *𝔹* is composed of the available *U* P300-labeled signals and the randomly selected *U* non-P300-labeled signals. We trained twice the number of *U* SVM by applying a leave-one-out cross-validation with the set *𝔹*. Then, we randomly selected one SVM. To evaluate the classifiers performance, we extracted a balanced subset *𝔹*′ from the validation set *𝕍*; the set *𝔹*′ is composed of the available *D* P300-labeled signals and the randomly selected *D* non-P300-labeled signals, where *D* = ⌊*P*′/*K*⌋ = 10. Then, we obtained the shape-feature vectors **v** (see Section 2.1) of  *𝔹*′. Finally, we classified such vectors with the selected SVM and with the SWLDA. We applied a confusion matrix to analyze the performance of both classifiers. The resulting accuracy *ψ* was computed by

(11) $$\begin{array}{c} \psi =\frac{\left(TP+TN\right)}{\left(TP+FP+FN+TN\right)}, \end{array}$$

where TP is the number of true positives, TN is the number of true negatives, FP is the number of false positives, and FN is the number of false negatives.

**Figure 8:**
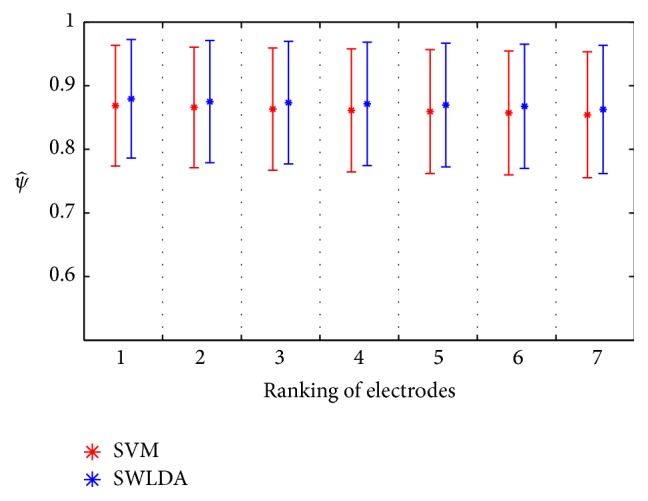
Ranking of electrodes according to the average accuracy of classifiers SWLDA and SVM for the studied population, where electrode 1 corresponds to the performance of the selected features, which includes information from all the electrodes, computed with the template of the best performing electrode for each subject (not necessarily the same), while electrode 7 is the one with the lowest *ψ* per subject.

**Figure 9:**
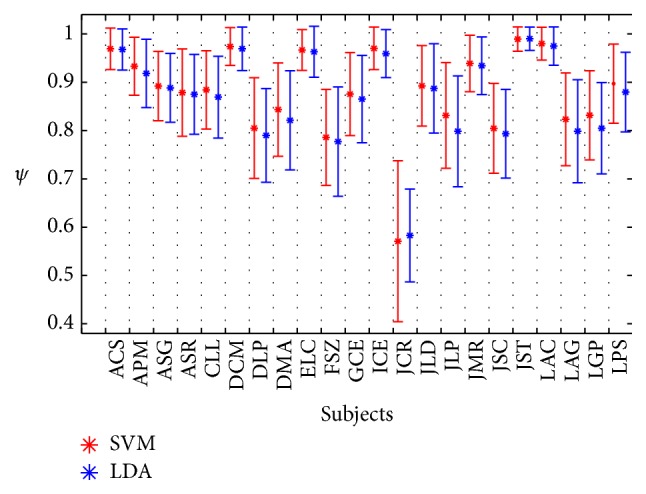
Detailed accuracy *ψ* of classifiers SVM and SWLDA for the electrode 1 of Figure 8 by using our shape-feature vector.

The average accuracy for the studied population $\left(\hat{\psi }\right)$ of both classifiers is presented in Figure 8, where “electrode 1” corresponds to the performance of the selected features, which includes information from all the electrodes, computed with the template of the best performing electrode for each subject (not necessarily the same), while “electrode 7” is the one with the lowest *ψ* per subject. In this figure, we can observe that the average value of “electrode 1” with the SVM for all subjects was 0.87 ± 0.09 and with the SWLDA was 0.88 ± 0.09. In addition, Figure 9 shows the detailed information yielding the accuracy for “electrode 1” for every subject: one of them had one electrode with *ψ* = 0.99 ± 0.02 with both classifiers; eight subjects had at least one electrode with *ψ* ≥ 0.9; 17 subjects had at least one electrode with *ψ* ≥ 0.8 with the SVM and 20 with the SWLDA; and 20 subjects had at least one electrode with *ψ* ≥ 0.7 with both classifiers. The worst case was one subject whose best *ψ* was equal to 0.59 ± 0.10 with the SVM and 0.57 ± 0.18 with the SWLDA, with distraction due to fatigue, lack of motivation, or hunger being a possible cause for such low accuracy. As stated in Section 3.1, the average value of the best electrode for all subjects was 0.87 ± 0.10, which is not different from the average value of “electrode 1” classified with the SVM, and with the SWLDA the average and the standard deviation have a difference of 0.01.

**Figure 10:**
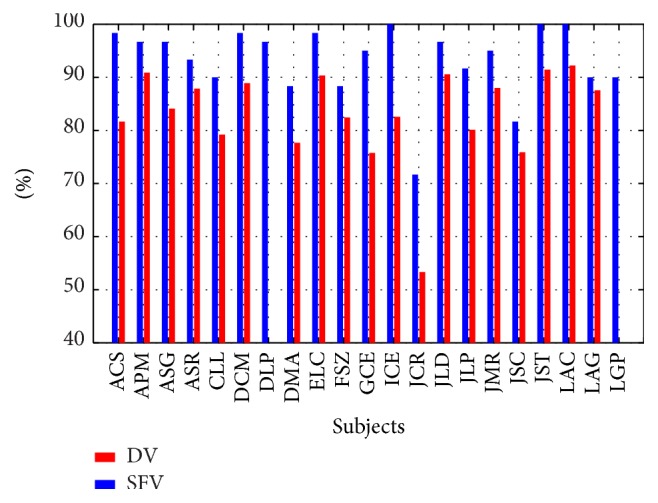
Comparison between the percentage of correct P300 detection by using the shape-feature vector (SFV) and the vector used by BCI2000 system (DV).

In order to evaluate the second experiment, we used an unseen unbalance set *𝕍*. First, we obtained its shape-feature vectors **v** (see Section 2.1) and its feature vectors **v**′ as used in the BCI2000 system. Then, we classified those vectors by the SWLDA. Since the set *𝕍* was labeled, we computed the percentage of correct classification. The SWLDA was unable to generate useful coefficients with the given parameters for two subjects when using the feature vectors **v**′. In contrast, it was able to generate weights for all the subjects when using the shape-feature vectors **v**. Thus, taking into account the nineteen subjects that both vectors could solve, the P300 detection using the SWLDA with the shape-feature vectors **v** was 93.15% ± 7.17, whereas with feature vectors **v**′ the detection was 83.18% ± 9.03. For all the subjects, the percentage of correct classification with the shape-feature vectors **v** was 10% higher than that with the feature vectors **v**′ (see Figure 10).

On the other hand, we evaluated the dimensionality reduction. We selected the elements of the shape-feature vector by the stepwise regression method. We observed that the SRM computes the maximum size of a vector (equal to 38 per electrode), because no additional terms satisfy the entry and removal criteria. However, it is possible to reduce even further the dimensionality of the vector while keeping an accuracy of one at least for one electrode; that was the case for nine subjects using the SWLDA and eight subjects using the SVM.

Finally, we compare other methods with ours. As mentioned earlier, the idea that a P300 is more similar to a template whose waveform resembles that of a P300 than to a non-P300 is not new. As mentioned in Section 1, there is a group of algorithms implementing* template matching classifiers* that can be used for detecting P300s (i.e., ML [13], DTW [14, 15], and Woody [12]). We consider our method as a member of this group. Our method is similar to DTW since both are based on slopes; however, our method is based on representing a signal waveform by a chain code. Additionally, unlike the methods based on an artificial template (such as those based on Woody's method [48]), we generate a template for every subject based on their own ERP signals by means of a* wrapping* method.

## 4. Conclusions

The P300 is an ERP elicited after the presentation of an infrequent stimulus. This endogenous component possesses some useful properties that permit controlling BCI applications [49]. For these applications to run in real time, it is important to optimize their computational resources. One indirect way to diminish the computational time is by reducing the dimensionality of the input feature vector used in the classification process without weakening the detection accuracy. The dimensionality reduction of such a vector can be achieved by lowering the number of electrodes used to acquire a subject's EEG. On the other hand, to improve the detection of a P300, several researchers have proposed representing this signal in different domains such as time, frequency, time-frequency, or shape. In this work, we have chosen the latter because we assume that a subject produces P300 signals whose waveform can be consistently represented by template curves and that these template curves are more similar to curves with a P300 than to curves produced by EEG background activity. The novelty of this work is the description of all these curves by means of their shape features. These features are represented as a vector of characteristics whose elements are provided by an adapted version (developed by us) of the Slope Chain Code. The latter is the most useful chain code for the purposes of this work because it divides the curve into straight-line segments placed onto the curve and preserves with higher resolution the contour shape. However, our chain code is computationally less expensive than the Slope Chain Code and, therefore, it is very useful for real-time applications. Similar to other chain codes, ours does not require decoding because it is self-contained and allows using grammatical and syntactic analysis techniques. In addition to the chain, we included in our vector other shape features such as the tortuosity measure (i.e., one of the curve's properties measured by the Slope Chain Code), the individual difference between the areas of every segment that divides the curve, and the sum of these differences.

In order to demonstrate our main hypothesis that our shape-feature vector improves the P300 detection accuracy, we designed some experiments which demonstrated that the performance of the SWLDA classifier is better when applying our feature vector than when applying the one used in the BCI2000 system [5]; our experiments also suggested that it is possible to significantly reduce the dimensionality of our feature vector while preserving a high accuracy during classification.

Because calibration is a crucial step of any BCI system, we have proposed a calibration methodology that achieves the following goals: (i) it obtains a set of templates that best represents, for a given electrode, the subject's P300 based on his/her own acquired signals, (ii) it finds the optimal number of trials for every subject, (iii) it selects the subset of electrodes that provides the best P300 signal for every subject, and (iv) it selects the shape features that maximize the classification accuracy while reducing the dimensionality of the feature vector. Our statistical tests showed that our method achieves a high average accuracy in the detection of P300 signals with fewer than fifteen stimulations. Furthermore, in agreement with the literature [5, 47], our results show that the best information is provided by the electrodes selected in the central, frontal, parietal, and parieto-occipital areas of the scalp.

Our future work will focus on the implementation of our methodology to a BCI. Additionally, we are planning further studies to analyze the robustness of the computed templates over time. Because there is evidence that the use of grammatical techniques and syntactic analysis yields promising results, we plan to investigate these techniques for detecting the P300 using the chain code approach. Finally, our chain code can be easily implemented on integer-number arithmetic; this makes it suitable for an efficient hardware implementation that integrates a classifier into signal acquisition devices, something that we are currently exploring.

## Figures and Tables

**Algorithm 1:**
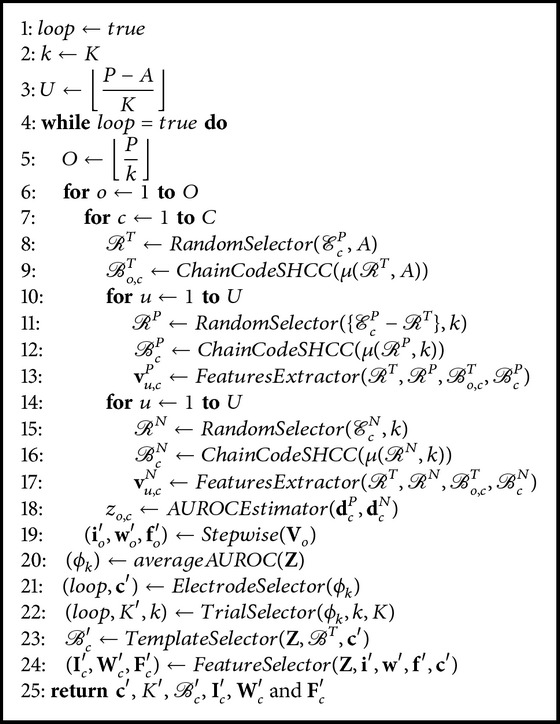
Calibration algorithm.

**Table 1:** Optimum number of stimulations. The middle column shows the best electrode for each subject.

Subjects	Electrodes	Stimulations
ACS	Cz	7
APM	Fz	7
ASG	PO8	5
ASR	Fz	15
CLL	PO8	15
DCM	PO8	2
DLP	Cz	15
DMA	C4	8
ELC	Pz	5
FSZ	Pz	15
GCE	PO8	5
ICE	Cz	4
JCR	Oz	15
JLD	Cz	9
JLP	Cz	15
JMR	PO8	4
JSC	Fz	9
JST	PO7	3
LAC	PO7	4
LAG	PO8	15
LGP	PO7	14
